# Recent Developments in Production and Biotechnological Applications of C-Phycocyanin

**DOI:** 10.1155/2013/742859

**Published:** 2013-08-26

**Authors:** M. Kuddus, P. Singh, G. Thomas, Awdah Al-Hazimi

**Affiliations:** ^1^Department of Biochemistry, College of Medicine, University of Hail, Hail 2440, Saudi Arabia; ^2^Department of Molecular and Cellular Engineering, SHIATS, Allahabad 211007, India

## Abstract

An extensive range of pigments including phycobiliproteins are present in algae. C-phycocyanin (C-PC), a phycobiliprotein, is one of the key pigments of *Spirulina*, a microalgae used in many countries as a dietary supplement. Algal pigments have massive commercial value as natural colorants in nutraceutical, cosmetics, and pharmaceutical industries, besides their health benefits. At present, increasing awareness of harmful effects of synthetic compounds and inclination of community towards the usage of natural products have led to the exploitation of microalgae as a source of natural pigments/colors. This review describes recent findings about the sources and production of C-PC, with emphasis on specific techniques for extraction and purification, along with potential industrial applications in diagnostics, foods, cosmetics, and pharmaceutical industries.

## 1. Introduction

In the quest of revealing nature's secrets during the past centuries, science has explored many concealed natural resources, thus opening a big market to the chemical industries. The chemicals used in the early ages were much expensive and caused biohazards. Thus, researches in finding safe, less-expensive, and easy-to-get natural bioactive compounds have been started. Since the advent of the analytical fields new assays and methods have been on the rise, thereby making the diagnostic industry a source of in-depth information. In the early period, the immunodiffusion assays provide information of remarkable magnitude followed by radio-immunoassays and enzyme-linked immunoassays. In these assays, different fluorochromes were exploited, and today it forms a separate field of interest as fluorescent immunoassay [1]. However, the synthetic fluorochromes cause biohazards and are carcinogenic in nature such as synthetic dye ethidium bromide. Thus a new approach of research was needed to find out novel chemicals which are not that expensive and are safe to handle. Phycobiliproteins are a group of colored proteins commonly present in cyanobacteria and red algae possessing a spectrum of applications. They are extensively commercialized for fluorescent application in clinical and immunological analysis. They are also used as colorant, and their therapeutic value has also been positively demonstrated [2]. Phycobiliproteins are brilliantly colored, highly fluorescent, water-soluble protein components of the photosynthetic light-harvesting antenna complexes of cyanobacteria (blue-green algae), red algae, and cryptomonads. These proteins are classified into two large groups based on their colors, the phycoerythrin (red), and the phycocyanin (blue). The phycocyanins include C-phycocyanin (C-PC), R-phycocyanin (R-PC), and allophycocyanin. Phycobiliproteins are assembled into an organized cellular structure, namely, the phycobilisomes that are attached in regular arrays to the external surface of the thylakoid membrane and act as major light-harvesting pigments in cyanobacteria and red algae. The term “phycobilisome” was first coined by Gantt and Conti [3] on the basis of their size and shape as visualized by electron microscope. The electron micrographs showed a series of large granules aligned regularly on the thylakoid membranes of different cyanobacteria and red alga, which were about twice the size and of similar shape to that of ribosomes; and attracted the attention of early biologists due to their brilliant colors. Phycobilisomes consist of allophycocyanin cores surrounded by phycocyanin on the periphery. Phycocyanin is the major constituent while allophycocyanin functions as the bridging pigment between phycobilisomes and the photosynthetic lamella [4]. The phycobilisomes allow the pigments to be arranged geometrically in a manner which helps to optimize the capture of light and transfer of energy. All the phycobiliproteins absorb incident light directly, but in addition they participate in an energy transfer chain within the phycobilisome in a sequence: phycoerythrin to phycocyanin to allophycocyanin to chlorophyll-a.

The phycobiliproteins were introduced as a novel class of fluorescent dyes in 1982 [5]. These naturally occurring fluorescent phycobiliproteins was used instantly in diagnostic assays and in diverse research applications [6, 7]. The phycobiliproteins serve as valuable fluorescent tags with numerous applications in flow cytometry, fluorescence activated cell sorting, histochemistry, and to a limited degree in immunoassay and detection of reactive oxygen species. These applications exploit the unique physical and spectroscopic properties of phycobiliproteins [8]. In addition, because of the high molecular absorptivity of these proteins at visible wavelengths, they are convenient markers in such applications as gel electrophoresis, isoelectric focusing, and gel exclusion chromatography [9, 10].

C-phycocyanin belongs to a family of phycobiliproteins that are well suited as a fluorescent reagent for immunological analysis, because they have a broad excitation spectrum and large stokes shift and fluoresce with a high quantum yield. C-PC is an antenna pigment used by mainly cyanobacteria and eukaryotic algae to increase efficiency of photosynthesis by collecting light energy at wavelength over which chlorophyll absorbs poorly. Phycobiliproteins are easily isolated as pigment protein complex, which is soluble in water and is very fluorescent [11]. It is a stable protein and contains multiple chromophore prosthetic groups, which are responsible for the fluorescent properties of this protein. They are attached to the surrounding protein structure via thioether linkage involving cystein residues. Chromophore prosthetic groups are constructed from linear or open tetrapyrrole ring and are structurally related to the bile pigment biliverdin. The four main chromophore types present in algal and cyanobacterial species are phycocyanobilin (PCB), phycoerythrobilin (PEB), phycourobilin (PUB), and cryptoviolin, whereas C-PC has only phycocyanobilin. The principal phycobiliproteins present in *Spirulina platensis* are phycocyanin and allophycocyanin which are made up of dissimilar *α* and *β* polypeptide subunits.

## 2. Sources of C-Phycocyanin and Its Production

The cyanobacteria, namely, *Spirulina* (renamed as *Arthrospira*), has been commercialized in several countries for its use as health food and for therapeutic purposes due to its valuable constituents particularly proteins and vitamins [12, 13]. It is also a rich and inexpensive source of the pigment like phycocyanin [14, 15]. The purification procedures for phycocyanin from crude algae extracts are usually obtained by a combination of different techniques such as ammonium sulfate precipitation, ion-exchange chromatography, and gel filtration chromatography to get pure phycocyanin. The cyanobacteria are potential source of C-PC pigments along with rhodophyta (Table 1). Their cultivation without organic substrates can be an economical advantage over the other microorganisms, and an optimized production of relevant compounds under controlled conditions is conceivable [16]. The various aspects of C-PC production have been reported by Eriksen [17]. According to the above report, production of C-PC includes four different options, namely, photoautotrophic, mixotrophic, heterotrophic, and recombinant production. The important features of these methods are presented in Table 2.

### 2.1. Photoautotrophic Production

This is an outdoor method of C-PC production by photoautotrophic cultures of cyanobacterium grown in open ponds predominantly at tropical and subtropical locations [56–58]. *Spirulina platensis *(*Arthrospira platensis*) has been commonly chosen as a host for C-PC production because of its availability rather than due to particular qualities of C-PC. It is among a few photoautotrophic microorganisms that can be grown in open ponds without being outcompeted by contaminating organisms, although contaminants do appear in open *A. platensis* cultures [59, 60]. The worldwide production of *A. platensis* has been increasing since 1980 [61, 62]. The majority of *A. platensis* dry weight (>3000 metric ton) that are produced worldwide annually are used for health products and animal feed additives [62, 63].

### 2.2. Mixotrophic Production

The mixotrophic cultivation of blue-green alga along with *A. platensis* is to be carried out in an enclosed reactor. Marquez et al. [64] found that the specific growth rate of mixotrophic cultures grown on glucose corresponds to the sum of the photoautotrophic and heterotrophic specific growth rates. The mixotrophic cultivation results in faster growth and increased maximal biomass concentrations compared to photoautotrophic cultures [64–66]. The comparative data of Eriksen [17] on the productivities of C-PC contents also showed a higher growth rate in the mixotrophic indoor cultures than in the photoautotrophic outdoor cultures of *A. platensis*.

### 2.3. Heterotrophic Production

The heterotrophic microbial processes are not limited by incident light intensities and have much higher production potentials than light-dependent processes. They are easier to scale up with regards to reactor size, mixing, gas transfer, productivity, and axenicity since high surface-to-volume ratios are not mandatory. The unicellular rhodophyte, *Galdieria sulphuraria*, is a candidate for heterotrophic production of C-PC. *G. sulphuraria* contains a major amount of C-PC and minor amount of allophycocyanin. Its natural habitat is hot, acidic springs, so the optimal growth conditions are found at temperatures above 40°C, and it is able to utilize a variety of carbon sources [67]. The properties of C-PC from heterotrophic *G. sulphuraria* resemble those of C-PC from other cyanobacterial sources. Schmidt et al. [68] and Graverholt and Eriksen [69] investigated the growth and C-PC production by *G. sulphuraria* 074G strains. The *Arthrospira* strains can also grow heterotrophically on glucose and fructose in darkness. However, the heterotrophic production of C-PC in *A. platensis* is not a viable option since the reported specific growth rates and pigment contents in heterotrophic *Arthrospira* strains are very low [66, 70].

### 2.4. Recombinant Production

Recombinant protein production is an option for heterotrophic synthesis of C-PC. Production of this multichain holoprotein phycobiliprotein is more challenging than production of other recombinant proteins. Complete synthesis of recombinant phycobiliprotein depends on coexpression of *α*- and *β*-chains as well as parallel synthesis and insertion of the correct phycobilin chromophores. Recombinant C-PC, in which *cpcA* and *cpcB* genes were fused to His_6_ tags for affinity chromatographic purification, has been produced in photoautotrophic *Anabaena* species, which naturally synthesize and insert phycocyanobilin into C-PC [71]. These fusion proteins were expressed as stable C-PC complexes. Coding sequences for different biospecific recognition domains were also fused to stabilized C-PC fusion constructs, and the expressed multidomain fusion proteins were used as fluorescent probes [17]. The gene engineering have resulted in the production of recombinant C-PC with novel functions. In heteromorphic hosts, recombinant holo-C-PC *α*-subunit has been expressed in *Escherichia coli*. The His_6_ tags have allowed purification by affinity chromatography, and domains with affinities for specific biological structures have been incorporated [72, 73].

## 3. Isolation of C-Phycocyanin

The methods of C-PC isolation are reported by various authors and summarized in a review on the subject [2]. The process of isolation involves various steps, namely, breakage of cell wall and cell disruption, primary isolation, purification, drying, and characterization of the end products. According to the nature of organisms, a variety of physical and chemical methods are encountered for cell disruption. Physical methods like sonication, cavitation, osmotic shock, and repeated freeze thawing are commonly encountered. In chemical methods, usage of acids, alkali, detergents, enzymes, and their combination thereof are reported. In general, combinations of a variety of physical and chemical methods are exploited for cell breakage. After cell breakage, clarification by centrifugation was performed, and the product is primarily isolated from the supernatant. It comprises fractionation using ammonium sulphate, dialysis, and polyethylene glycol precipitation. Further purification is usually achieved by column chromatographic methods using adsorbents, molecular sieves, anion exchangers, or their combinations. According to the nature of organisms, a combination of physical and chemical methods with primary isolation and purification of the product is adapted. For drying the pigment, only freeze drying was found to be suitable. It is up to the technologist to choose any of these methods and their combination to achieve maximum recovery of the product. The characterization of phycobiliproteins including C-PC is required for the molecular understanding on various structures of phycobilisomes, and this provides a basis for the modeled reconstruction of the pigment complex. In order to exploit these natural colored substances, the C-PC is to be extracted from the phycobilisome and purified. Extraction of the phycobiliprotein from cyanobacteria and microalgae is very difficult because of the extremely resistant cell wall and the small size of the bacteria [74, 75]. However, various methods can be employed for extraction of phycobiliproteins, but no standard technique to quantitatively extract the C-PC pigment from microalgae exists [76]. A method that works well in one organism may not be the method of choice for another organism [77].

C-phycocyanin has been extracted by resuspending the biomass in 0.1 M phosphate buffer, pH 7.0, [39, 78] or 0.5 M (NH_4_)_2_SO_4_ [40]. Doke [39] found that most C-PC could be extracted from biomass dried at low temperatures. At 25°C, 80 mg C-PC per gram dried biomass could be extracted compared to just 16.5 mg g^−1^ from biomass dried at 50°C. Oliveira and coworkers [78] found that high drying temperatures in two different dryers decreased the amount of extractable C-PC from *A. platensis*. From wet biomass, C-PC has been extracted by subjecting the biomass to cycles of freezing at −25 to −15°C or in liquid nitrogen and thawing at 4 to 30°C [32, 35, 39, 41, 53]. When compared to alternative methods, freeze-thaw cycles have been the most efficient way to extract C-PC from wet cyanobacterial biomass [39, 53]. C-PC has also been extracted after mechanical cell disruption [42, 68], lysozyme treatment [42], sonication [53, 79], and high pressure exposure [43, 80]. It has also been described that live *Klebsiella pneumonia* effectively lyses *A. platensis* and extracts C-PC in 24 hours [44].

## 4. Purification and Characterization of C-Phycocyanin

In light of the considerable commercial application particularly as fluorescence tags, purity of the C-PC pigments plays a major role. The purity of C-PC preparations is evaluated based on the ratio between absorbencies from phycocyanobilin at 620 nm (*A*_620_) and aromatic amino acids in all proteins in the preparation at 280 nm (*A*_280_). C-PC preparations with *A*_620_/*A*_280_ greater than 0.7 were considered food grade as described by Rito-Palomares et al. [10], while *A*_620_/*A*_280_ of 3.9 were reactive grade and *A*_620_/*A*_280_ greater than 4.0 analytical grade. Herrera et al. [36] combined ultrafiltration, charcoal adsorption, and spray drying to obtain C-PC with *A*_620_/*A*_280_ of 0.74 and a yield of 34%, while additional chromatographic steps were included to purify C-PC to *A*_620_/*A*_280_ of 3.91 with a yield of 9%. Ammonium sulphate precipitation combined with a variety of chromatographic principles has been employed to obtain C-PC of food, reactive, and analytical grades [21, 22, 32, 35, 40–42, 53]. Also two-phase aqueous extraction was developed into an efficient method for C-PC purification [10] which resulted in highly pure C-PC preparations and high yields [43, 80]. Two-phase aqueous extraction followed by ion-exchange chromatography was recently reported to result in extremely pure C-PC with *A*_620_/*A*_280_ value of 6.69 [33]. 

C-phycocyanin is composed of *αβ* heterodimers, each heterodimer containing three linear tetrapyrrole chromophores, referred to as phycocyanobilins. Three *αβ* units oligomerize as disc-shaped trimers (*αβ*)_3_ which in turn form hexamers (*αβ*)_6_, and these hexamers stack one above the other forming the rod-like structure of phycobilosome [81]. The molecular architectures of these photosynthetic complexes are rapidly becoming available through the power of X-ray crystallography. Twelve crystal structures of phycocyanins were determined with an ultimate goal to obtain a physical picture of energy absorption and transfer through the complex to the reaction center. The C-PC structure has been determined by X-ray crystallography at a resolution of 1.45 Å [51]. Adir [82] analyzed the molecular understanding on various structures of phycobilisomes and suggested that the structural information obtained from the components provides the basis for the modeled reconstruction of this pigment complex.

## 5. Applications of C-Phycocyanin

The growing awareness on the importance of natural colors, especially in food and cosmetics colorants, has placed great demand on biological sources of natural colors. Cyanobacteria possess a wide range of colored components including carotenoids, chlorophyll, and phycocyanin [83]. It has gained importance in the development of phycofluor probes for immunodiagnostics [84]. C-phycocyanin is commonly used as natural dye in food and cosmetics and replaced the synthetic dyes. Phycocyanin has a significant antioxidant, anti-inflammatory, hepatoprotective, and radical-scavenging properties, it is even used in food coloring and in cosmetics, as it is nontoxic and non-carcinogenic [85]. The main application of phycobiliproteins is as fluorescent markers of cells and macromolecules in biomedical research and highly sensitive fluorescence techniques [8]. Because of their properties, C-PCs have been used in a variety of immunological assays and as fluorescent labels for cell sorting, in addition, because of the high molar absorptivity of C-PC and other phycobiliproteins at visible wavelength, they are convenient markers in such applications as gel electrophoresis, isoelectric focusing, and gel exclusion chromatography. This has further enhanced the scope of the diagnostic industry. C-PC can be used for the detection of multiple myeloma cells. It is used as a potential therapeutic agent in oxidative stress induced disease [86]. The pharmaceutical industry demands highly pure C-PC with absorption ratio (*A*_620_/*A*_280_) of 4 and food industry a ratio of 2 [87]. 

### 5.1. C-Phycocyanin as a Natural Dye

C-phycocyanin is the major phycobiliprotein in many blue-green algae. The intense blue color in blue-green algae is due to the presence of phycocyanin and emits red fluorescence. The pigment has a single visible absorbance maximum between 615 and 620 nm. It has maximum fluorescence emission at around 640 nm with molecular weight in between 70 and 110 kD. The pigment is composed of two subunits of *α* and *β* which occur in equal numbers. However, the exact number of *αβ* pair may vary among different species. Both *α* and *β* subunits contain only the phycocyanobilin chromophore. In addition to absorbing light directly, this intensely blue pigment accepts quanta from phycoerythrin by fluorescent energy transfer in organisms in which phycoerythrin is present. Also the C-PC pigment is widely used as natural dye for various purposes due to its deep and intense blue color. They are well suited as fluorescent reagent without any toxic effect for immunological analysis since they have a broad excitation spectrum and fluorescence with a high quantum yield [88]. They can be used as a valuable fluorescent probe for analysis of cells and molecules [89].

### 5.2. C-Phycocyanin as Food Additives and Health Foods

The deep blue colored phycocyanin and other extractable pigments including myxoxanthophyll and zeaxanthin extracted from microalga *Spirulina* have been used as naturally occurring colorant for food additive purposes [14, 90]. A few studies have addressed the functionality of C-PC in foods with regards to color stability [91, 92] and rheological properties [93]. C-PC from *A. platensis* is marketed as a food and cosmetics in Japan [94], whereas this has not been approved as such in the European Union. Limited customer preference in consuming blue foods might have probably minimized the industries interest in coloring the food with C-PC [17]. More attention has been paid on the use of C-PC as a nutraceutical particularly in health foods in which dried *A. platensis* is the functional component.

### 5.3. Diagnostic Applications of C-Phycocyanin

When phycobilisomes are extracted into aqueous buffers, they disintegrate and the phycobiliproteins lose their natural acceptors of excitation energy and become highly fluorescent. Compared to other fluorophores, phycobiliproteins have high molar extinction coefficient and fluorescence quantum yield and large stoke shifts. The apoprotein chain contains amino and carboxyl groups that can form bonds to other molecules [8, 95, 96]. Phycobiliproteins conjugated to immunoglobins, protein A, and avidin were developed into fluorescent probes and have obtained wide usage in histochemistry, fluorescence microscopy, flow cytometry, fluorescence-activated cell sorting, and fluorescence immunoassays [2, 95, 96]. The high molar extinction coefficients are the result of many chromophores per phycobiliprotein complex and are therefore higher for *α*_6_ *β*_6_ hexamers and *α*_3_ *β*_3_ trimers than *αβ* monomers. Also the extinction coefficients of individual phycobilins decrease when hexamers disintegrate into trimmers, and monomers [97]. The fluorescence quantum yield decreases when phycobiliprotein complexes dissociate and the chromophores gain increased conformational freedom. Finally, the extinction coefficients are diminished and fluorescence is almost lost when the phycobiliproteins are getting denatured [98].

The C-PC is isolated as mixtures of hexamers, trimers and monomers and has a quantum yield of 50% [5]. When C-PC is dissolved in dilute phosphate buffer at concentrations below 1 M or 30 mM, the monomers will be dominant and the fluorescence yield low [96, 98]. The use of C-PC in fluorescent probes is dependent on chemical cross linking of peptides to form stable trimers [99]. Absorbance and fluorescence emission spectra of the chemically stabilized C-PC trimers are very similar to native C-PC trimers, except that their coefficients are actually increased. These chemically stabilized C-PC trimers can be used as fluorescent probes with spectral properties different from other phycobiliproteins. Also complete phycobilisomes from *A. platensis* composed of C-PC have been chemically stabilized, combined to streptavidin, and used as fluorescent probes in cytometry [100]. As described previously, also genetically stabilized C-PC fusion proteins fused to biospecific recognition domains have been used directly as biospecific fluorescent probes [17, 71]. The other applications of phycocyanins as fluorescent probe includes the utilization of *in vivo* fluorescence from phycocyanin for online monitoring of growth in cyanobacterial cultures [101], detection of toxic cyanobacteria in drinking water [102], and remote sensing of cyanobacteria in natural water bodies [103]. Badakere et al. [104] and Kulkarni et al. [89] reported the possibility of staining RBCs, WBCs, platelets, lymphocytes, nucleated cells, and genomic DNA with C-PC. Singh et al. [49] also concluded that the partially purified C-PC could be used as a substitute of ethidium bromide and may be applied for immunological analysis and DNA staining.

### 5.4. Nutraceutical and Pharmaceutical Applications

Purified C-PC has nutraceutical and pharmaceutical potentials [12, 105]. A variety of impaired physiological conditions are reported to be relieved by C-PC administration [106–108]. It has also been observed that C-PC can inhibit cell proliferation [106], induce apoptosis in cancerogenic cell lines [109], and affect gene regulation in mammalian cell lines [107]. The antioxidant and radical-scavenging activities of C-PC from different cyanobacteria are well documented [33, 85, 86, 109–111]. Enhanced radical-scavenging activities have been reported in selenium-enriched C-PC obtained from *A. platensis* grown in Se-enriched medium [45, 112]. However, intact C-PC may not be the dominant functional antioxidants *in vivo.* McCarty [113] proposed that it is phycocyanorubin, a reduced form of phycocyanobilin, which is an important antioxidant species *in vivo* based on its similarity to bilirubin. Bilirubin is a natural antioxidant in plasma [15, 113] which inhibits formation of superoxide radicals by NADPH oxidase and may therefore play additional protective roles by reducing the generation of reactive oxygen species in the body. These observations have launched a further interest in C-PC as a nutraceutical or pharmaceutical with anticarcinogenic and other possible health effects. Also recombinant apo-C-PC *β*-subunit has been observed to inhibit cell proliferation and cause apoptosis in carcinoma cells [114]. One of the recent findings indicates that CPC could be potentially useful for treatment of LPS-related acute lung injury by inhibiting inflammatory responses and apoptosis in lung tissues [115].

## 6. Future Scenario

Cyanobacterial biomass has been considered as source of protein besides its nutritional value. The cyanobacteria are also a potential source of C-PC. Their cultivation under controlled conditions and low-cost downstream processing would be an economical advantage to fulfill their current requirement. Among the various factors affecting productivity and composition of pigment, light intensity and quality have high significant value. For the extraction of proteins from algal cells, a specific procedure should be followed depending on the nature of cells. The commercial potential of C-PC has some major obstacles such as widespread utilization and increasing the product yield. These problems may be solved through linkages between laboratory researchers and industrial technologists. The various approaches directed towards low-cost production and harvesting technologies along with evaluation of novel environmental conditions for algal production may be useful. This review brings out recent developments in production and applications of C-PC. However, efforts have to be made in order to achieve economical overproduction of C-PC by recombinant DNA technology and increase its nutritional and pharmacological values by protein engineering.

## Figures and Tables

**Table 1:** C-phycocyanin producing cyanobacterial strains.

C-PC producing organisms	Reference
*Anabaena marina *	[18]
*Anabaena* sp.	[19, 20]
*Aphanizomenon flos-aquae *	[21]
*Arthronema africanum *	[22]
*Coccochloris elabens *	[23]
*Cyanidium caldarium *	[24–26]
*Galdieria sulphuraria *	[27]
*Gracilaria chilensis *	[28]
*Lyngbya* sp.	[29]
*Mastigocladus laminosus *	[30]
*Microcystis *	[31]
*Nostoc, Phormidium *	[20]
*Oscillatoria quadripunctulata *	[32]
*Phormidium fragile *	[33, 34]
*Spirulina fusiformis *	[35]
*Spirulina maxima *	[10, 36–38]
*Spirulina platensis/Arthrospira platensis *	[39–48]
*Spirulina* sp.	[49]
*Synechocystis* sp.	[50]
*Synechococcus elongates *	[51]
*Synechococcus lividus *	[52]
*Synechococcus vulcanus *	[53, 54]
*Synechococcus* sp.	[55]

**Table 2:** Different method of C-PC production.

Sl Number	Method	Characteristics
1	Photoautotrophic production	An out-door method of C-PC production by using open ponds. Mostly *A. platensis* is used for dry weight production.

2	Mixotrophic production	Production is carried out in an enclosed reactor. Specific growth rate of mixotrophic cultures corresponds to sum of photoautotrophic and heterotrophic specific growth rates. Higher growth rate of *A. platensis* is found in mixotrophic indoor cultures compared to photoautotrophic outdoor cultures.

3	Heterotrophic production	Production is not limited by incident light intensity. The unicellular rhodophyte, *Galdieria sulphuraria*, is a candidate for heterotrophic production of C-PC.

4	Recombinant production	Recombinant protein production is an option for heterotrophic synthesis of C-PC. Recombinant C-PC has been produced in photoautotrophic *Anabaena*.

## References

[1] Kasten FH (1993). Introduction to fluorescent probes: properties, history and applications. *Fluorescent and Luminescent Probes for Biological Activity*.

[2] Sekar S, Chandramohan M (2008). Phycobiliproteins as a commodity: trends in applied research, patents and commercialization. *Journal of Applied Phycology*.

[3] Gantt E, Conti SF (1966). Granules associated with the chloroplast lamellae of *Porphyridium cruentum*. *Journal of Cell Biology*.

[4] Eisele LE, Bakhru SH, Liu X, MacColl R, Edwards MR (2000). Studies on C-phycocyanin from *Cyanidium caldarium*, a eukaryote at the extremes of habitat. *Biochimica et Biophysica Acta*.

[5] Oi VT, Glazer AN, Stryer L (1982). Fluorescent phycobiliprotein conjugates for analyses of cells and molecules. *Journal of Cell Biology*.

[6] Kronick MN (1986). The use of phycobiliproteins as fluorescent labels in immunoassay. *Journal of Immunological Methods*.

[7] Glazer AN, Stryer L (1990). Phycobiliprotein-avidin and phycobiliprotein-biotin conjugates. *Methods in Enzymology*.

[8] Glazer AN (1994). Phycobiliproteins—a family of valuable, widely used fluorophores. *Journal of Applied Phycology*.

[9] Sarada R, Pillai MG, Ravishankar GA (1999). Phycocyanin from *Spirulina* sp: influence of processing of biomass on phycocyanin yield, analysis of efficacy of extraction methods and stability studies on phycocyanin. *Process Biochemistry*.

[10] Rito-Palomares M, Nuez L, Amador D (2001). Practical application of aqueous two-phase systems for the development of a prototype process for C-phycocyanin recovery from *Spirulina maxima*. *Journal of Chemical Technology and Biotechnology*.

[11] Glazer AN (1982). Phycobilisomes: structure and dynamics. *Annual Review of Microbiology*.

[12] Belay A, Ota Y, Miyakawa K, Shimamatsu H (1993). Current knowledge on potential health benefits of *Spirulina*. *Journal of Applied Phycology*.

[13] Dillon JC, Phuc AP, Dubacq JP (1995). Nutritional value of the alga *Spirulina*. *World Review of Nutrition and Dietetics*.

[14] Kato T (1994). Blue pigment from *Spirulina*. *New Food Industry*.

[15] McCarty MF (2007). Clinical potential of *Spirulina* as a source of phycocyanobilin. *Journal of Medicinal Food*.

[16] Kreitlow S, Mundt S, Lindequist U (1999). Cyanobacteria—a potential source of new biologically active substances. *Journal of Biotechnology*.

[17] Eriksen NT (2008). Production of phycocyanin—a pigment with applications in biology, biotechnology, foods and medicine. *Applied Microbiology and Biotechnology*.

[18] Ramos A, Acién FG, Fernández-Sevilla JM, González CV, Bermejo R (2010). Large-scale isolation and purification of C-phycocyanin from the cyanobacteria *Anabaena marina* using expanded bed adsorption chromatography. *Journal of Chemical Technology and Biotechnology*.

[19] Moreno J, Rodríguez H, Vargas MA, Rivas J, Guerrero MG (1995). Nitrogen-fixing cyanobacteria as source of phycobiliprotein pigments. Composition and growth performance of ten filamentous heterocystous strains. *Journal of Applied Phycology*.

[20] Shukia SP, Singh JS, Kashyap S, Giri DD, Kashyap AK (2008). Antarctic cyanobacteria as a source of phycocyanin: an assessment. *Indian Journal of Marine Sciences*.

[21] Benedetti S, Rinalducci S, Benvenuti F (2006). Purification and characterization of phycocyanin from the blue-green alga Aphanizomenon flos-aquae. *Journal of Chromatography B*.

[22] Minkova K, Tchorbadjieva M, Tchernov A, Stojanova M, Gigova L, Busheva M (2007). Improved procedure for separation and purification of *Arthronema africanum* phycobiliproteins. *Biotechnology Letters*.

[23] Kao OHW, Berns DS, Town WR (1973). The characterization of C-phycocyanin from an extremely halo-tolerant blue-green alga, *Coccochloris elabens*. *Biochemical Journal*.

[24] Kao OHW, Edwards MR, Berns DS (1975). Physical chemical properties of C-phycocyanin isolated from an acido thermophilic eukaryote, *Cyanidium caldarium*. *Biochemical Journal*.

[25] Troxler RF, Ehrhardt MM, Brown-Mason AS, Offner GD (1981). Primary structure of phycocyanin from the unicellular rhodophyte *Cyanidium caldarium*. II. Complete amino acid sequence of the beta subunit. *Journal of Biological Chemistry*.

[26] Stec B, Troxler RF, Teeter MM (1999). Crystal structure of C-phycocyanin from *Cyanidium caldarium* provides a new perspective on phycobilisome assembly. *Biophysical Journal*.

[27] Graverholt OS (2004). *Production and characterization of phycocyanin in different isolates of Galdieria sulphuraria [M.S. thesis]*.

[28] Contreras-Martel C, Matamala A, Bruna C (2007). The structure at 2 Å resolution of Phycocyanin from *Gracilaria chilensis* and the energy transfer network in a PC-PC complex. *Biophysical Chemistry*.

[29] Patel A, Mishra S, Pawar R, Ghosh PK (2005). Purification and characterization of C-phycocyanin from cyanobacterial species of marine and freshwater habitat. *Protein Expression and Purification*.

[30] Kao OHW, Berns DS (1977). Similar C-phycocyanins from two strains of thermotolerant cyanophyte *Mastigocladus laminosus*. *Canadian Journal of Microbiology*.

[31] Wang CY, Wang X, Wang Y (2012). Photosensitization of phycocyanin extracted from *Microcystis* in human hepatocellular carcinoma cells: implication of mitochondria-dependent apoptosis. *The Journal of Photochemistry and Photobiology B*.

[32] Soni B, Kalavadia B, Trivedi U, Madamwar D (2006). Extraction, purification and characterization of phycocyanin from *Oscillatoria quadripunctulata*-isolated from the rocky shores of Bet-Dwarka, Gujarat, India. *Process Biochemistry*.

[33] Soni B, Trivedi U, Madamwar D (2008). A novel method of single step hydrophobic interaction chromatography for the purification of phycocyanin from *Phormidium fragile* and its characterization for antioxidant property. *Bioresource Technology*.

[34] Satyanarayana L, Suresh CG, Patel A, Mishra S, Ghosh PK (2005). X-ray crystallographic studies on C-phycocyanins from cyanobacteria from different habitats: marine and freshwater. *Acta Crystallographica Section F*.

[35] Minkova KM, Tchernov AA, Tchorbadjieva MI, Fournadjieva ST, Antova RE, Busheva MC (2003). Purification of C-phycocyanin from *Spirulina (Arthrospira) fusiformis*. *Journal of Biotechnology*.

[36] Herrera A, Boussiba S, Napoleone V, Hohlberg A (1989). Recovery of C-phycocyanin from the cyanobacterium *Spirulina maxima*. *Journal of Applied Phycology*.

[37] Muthulakshmi M, Saranya A, Sudha M, Selvakumar G (2012). Extraction, partial purification, and antibacterial activity of phycocyanin from *Spirulina* isolated from fresh water body against various human pathogens. *Journal of Algal Biomass Utilization*.

[38] Abd El-Baky HH, El-Baroty GS (2012). Characterization and bioactivity of phycocyanin isolated from *Spirulina maxima* grown under salt stress. *Food and Function*.

[39] Doke JH (2005). An improved and efficient method for the extraction of phycocyanin from *Spirulina* sp. *International Journal of Food Engineering*.

[40] Niu J-F, Wang G-C, Lin X-Z, Zhou B-C (2007). Large-scale recovery of C-phycocyanin from *Spirulina platensis* using expanded bed adsorption chromatography. *Journal of Chromatography B*.

[41] Zhang Y-M, Chen F (1999). A simple method for efficient separation and purification of C-phycocyanin and allophycocyanin from *Spirulina platensis*. *Biotechnology Techniques*.

[42] Boussiba S, Richmond AE (1979). Isolation and characterization of phycocyanins from the blue-green alga *Spirulina platensis*. *Archives of Microbiology*.

[43] Patil G, Chethana S, Sridevi AS, Raghavarao KSMS (2006). Method to obtain C-phycocyanin of high purity. *Journal of Chromatography A*.

[44] Zhu Y, Chen XB, Wang KB (2007). A simple method for extracting C-phycocyanin from *Spirulina platensis* using *Klebsiella pneumoniae*. *Applied Microbiology and Biotechnology*.

[45] Chen T, Zheng W, Yang F, Bai Y, Wong Y-S (2006). Mixotrophic culture of high selenium-enriched *Spirulina platensis* on acetate and the enhanced production of photosynthetic pigments. *Enzyme and Microbial Technology*.

[46] Duangsee R, Phoopat N, Ningsanond S (2009). Phycocyanin extraction from *Spirulina platensis* and extract stability under various pH and temperature. *Asian Journal of Food and Agro-Industry*.

[47] Walter A, de Carvalho J, Soccol VT, de Faria AB, Ghiggi V, Soccol CR (2011). Study of phycocyanin production from *Spirulina platensis* under different light spectra. *Brazilian Archives of Biology and Technology*.

[48] Mohite YS, Wakte PS (2011). Assessment of factors influencing growth and C-phycocyanin production of *Arthrospira platensis* from meteoritic Crater Lake. *Journal of Algal Biomass Utilization*.

[49] Singh P, Kuddus M, Thomas G (2010). An efficient method for extraction of C-phycocyanin from *Spirulina* sp. and its binding affinity to blood cells, nuclei and genomic DNA. *International Research Journal of Biotechnology*.

[50] Rogner M, Nixon PJ, Diner BA (1990). Purification and characterization of photosystem I and photosystem II core complexes from wild-type and phycocyanin-deficient strains of the *Cyanobacterium synechocystis* PCC 6803. *Journal of Biological Chemistry*.

[51] Nield J, Rizkallah PJ, Barber J, Chayen NE (2003). The 1.45 Å three-dimensional structure of C-phycocyanin from the thermophilic cyanobacterium *Synechococcus elongatus*. *Journal of Structural Biology*.

[52] MacColl R, Edwards MR, Mulks MH, Berns DS (1974). Comparison of the biliproteins from two strains of the thermophilic cyanophyte *Synechococcus lividus*. *Biochemical Journal*.

[53] Abalde J, Betancourt L, Torres E, Cid A, Barwell C (1998). Purification and characterization of phycocyanin from the marine cyanobacterium *Synechococcus* sp. IO9201. *Plant Science*.

[54] Adir N, Dobrovetsky Y, Lerner N (2001). Structure of C-phycocyanin from the thermophilic cyanobacterium *Synechococcus vulcanus* at 2.5 Å: structural implications for thermal stability in phycobilisome assembly. *Journal of Molecular Biology*.

[55] Gupta A, Sainis JK (2010). Isolation of C-phycocyanin from *Synechococcus* sp., (*Anacystis nidulans* BD1). *Journal of Applied Phycology*.

[56] Pulz O (2001). Photobioreactors: production systems for phototrophic microorganisms. *Applied Microbiology and Biotechnology*.

[57] Carlozzi P (2003). Dilution of solar radiation through “culture” lamination in photobioreactor rows facing south-north: a way to improve the efficiency of light utilization by cyanobacteria (*Arthrospira platensis*). *Biotechnology and Bioengineering*.

[58] Jiménez C, Cossío BR, Labella D, Niell FX (2003). The feasibility of industrial production of *Spirulina* (*Arthrospira*) in Southern Spain. *Aquaculture*.

[59] Richmond A, Grobbelaar JU (1986). Factors affecting the output rate of *Spirulina platensis* with reference to mass cultivation. *Biomass*.

[60] Richmond A, Lichtenberg E, Stahl B, Vonshak A (1990). Quantitative assessment of the major limitations on productivity of *Spirulina platensis* in open raceways. *Journal of Applied Phycology*.

[61] Borowitzka MA (1999). Commercial production of microalgae: ponds, tanks, tubes and fermenters. *Journal of Biotechnology*.

[62] Pulz O, Gross W (2004). Valuable products from biotechnology of microalgae. *Applied Microbiology and Biotechnology*.

[63] Spolaore P, Joannis-Cassan C, Duran E, Isambert A (2006). Commercial applications of microalgae. *Journal of Bioscience and Bioengineering*.

[64] Marquez FJ, Sasaki K, Kakizono T, Nishio N, Nagai S (1993). Growth characteristics of *Spirulina platensis* in mixotrophic and heterotrophic conditions. *Journal of Fermentation and Bioengineering*.

[65] Vonshak A, Cheung SM, Chen F (2000). Mixotrophic growth modifies the response of *Spirulina (Arthrospira) platensis* (Cyanobacteria) cells to light. *Journal of Phycology*.

[66] Chojnacka K, Noworyta A (2004). Evaluation of *Spirulina* sp. growth in photoautotrophic, heterotrophic and mixotrophic cultures. *Enzyme and Microbial Technology*.

[67] Gross W, Schnarrenberger C (1995). Heterotrophic growth of two strains of the acido-thermophilic red alga *Galdieria sulphuraria*. *Plant and Cell Physiology*.

[68] Schmidt RA, Wiebe MG, Eriksen NT (2005). Heterotrophic high cell-density fed-batch cultures of the phycocyanin-producing red alga *Galdieria sulphuraria*. *Biotechnology and Bioengineering*.

[69] Graverholt OS, Eriksen NT (2007). Heterotrophic high-cell-density fed-batch and continuous-flow cultures of *Galdieria sulphuraria* and production of phycocyanin. *Applied Microbiology and Biotechnology*.

[70] Mühling M, Belay A, Whitton BA (2005). Screening *Arthrospira* (*Spirulina*) strains for heterotrophy. *Journal of Applied Phycology*.

[71] Cai YA, Murphy JT, Wedemayer GJ, Glazer AN (2001). Recombinant phycobiliprotiens: recombinant C-phycocyanins equipped with affinity tags, oligomerization, and biospecific recognition domains. *Analytical Biochemistry*.

[72] Tooley AJ, Cai YA, Glazer AN (2001). Biosynthesis of a fluorescent cyanobacterial C-phycocyanin holo-*α* subunit in a heterologous host. *Proceedings of the National Academy of Sciences of the United States of America*.

[73] Guan X, Qin S, Zhao F, Zhang X, Tang X (2007). Phycobilisomes linker family in cyanobacterial genomes: divergence and evolution. *International Journal of Biological Sciences*.

[74] Stewart DE, Farmer FH (1984). Extraction, identification and quantitation of phycobiliprotein pigments from phototrophic plankton. *Limnology & Oceanography*.

[75] Wyman M (1992). An *in vivo* method for the estimation of phycoerythrin concentration in marine cyanobacteria (*Synechococcus* spp.). *Limnology & Oceanography*.

[76] Wiltshire KH, Boersma M, Möller A, Buhtz H (2000). Extraction of pigments and fatty acids from the green alga *Scenedesmus obliquus* (Chlorophyceae). *Aquatic Ecology*.

[77] Ranjitha K, Kaushik BD (2005). Purification of phycobiliproteins from *Nostoc muscorum*. *Journal of Scientific and Industrial Research*.

[78] Oliveira EG, Rosa GS, Moraes MA, Pinto LAA (2008). Phycocyanin content of *Spirulina platensis* dried in spouted bed and thin layer. *Journal of Food Process Engineering*.

[79] Furuki T, Maeda S, Imajo S (2003). Rapid and selective extraction of phycocyanin from *Spirulina platensis* with ultrasonic cell disruption. *Journal of Applied Phycology*.

[80] Patil G, Raghavarao KSMS (2007). Aqueous two phase extraction for purification of C-phycocyanin. *Biochemical Engineering Journal*.

[81] Glazer AN (1989). Light guides. Directional energy transfer in a photosynthetic antenna. *Journal of Biological Chemistry*.

[82] Adir N (2005). Elucidation of the molecular structures of components of the phycobilisome: reconstructing a giant. *Photosynthesis Research*.

[83] Gantt E (1975). Phycobilisomes: light harvesting pigment complexes. *BioScience*.

[84] Kronick MN, Grossman PD (1983). Immunoassay techniques with fluorescent phycobiliprotein conjugates. *Clinical Chemistry*.

[85] Romay C, González R (2000). Phycocyanin is an antioxidant protector of human erythrocytes against lysis by peroxyl radicals. *Journal of Pharmacy and Pharmacology*.

[86] Bhat VB, Madyastha KM (2000). C-phycocyanin: a potent peroxyl radical scavenger *in vivo* and *in vitro*. *Biochemical and Biophysical Research Communications*.

[87] Uday Bhaskar S, Gopalaswamy G, Raghu R (2005). A simple method for efficient extraction and purification of C-phycocyanin from *Spirulina platensis* Geitler. *Indian Journal of Experimental Biology*.

[88] Hardy RR, Weir DM (1986). Purification and coupling of fluorescent proteins for use in flow cytometry. *Handbook of Experimental Immunology*.

[89] Kulkarni SU, Badakere SS, Oswald J, Kamat MY (1996). Fluorescent phycocyanin from *Spirulina platensis*. Application for diagnosis. *Biotech Lab International*.

[90] Hirata T, Tanaka M, Ooike M, Tsunomura T, Sakaguchi M (2000). Antioxidant activities of phycocyanobilin prepared from *Spirulina platensis*. *Journal of Applied Phycology*.

[91] Jespersen L, Strømdahl LD, Olsen K, Skibsted LH (2005). Heat and light stability of three natural blue colorants for use in confectionery and beverages. *European Food Research and Technology*.

[92] Mishra SK, Shrivastav A, Mishra S (2008). Effect of preservatives for food grade C-PC from *Spirulina platensis*. *Process Biochemistry*.

[93] Batista AP, Raymundo A, Sousa I, Empis J (2006). Rheological characterization of coloured oil-in-water food emulsions with lutein and phycocyanin added to the oil and aqueous phases. *Food Hydrocolloids*.

[94] Prasanna R, Sood A, Suresh A, Nayak S, Kaushik BD (2007). Potentials and applications of algal pigments in biology and industry. *Acta Botanica Hungarica*.

[95] Glazer AN, Stryer L (1984). Phycofluor probes. *Trends in Biochemical Sciences*.

[96] Sun L, Wang S, Chen L, Gong X (2003). Promising fluorescent probes from phycobiliproteins. *IEEE Journal on Selected Topics in Quantum Electronics*.

[97] Thoren KL, Connell KB, Robinson TE, Shellhamer DD, Tammaro MS, Gindt YM (2006). The free energy of dissociation of oligomeric structure in phycocyanin is not linear with denaturant. *Biochemistry*.

[98] Kupka M, Scheer H (2008). Unfolding of C-phycocyanin followed by loss of non-covalent chromophore-protein interactions. 1. Equilibrium experiments. *Biochimica et Biophysica Acta*.

[99] Sun L, Wang S, Qiao Z (2006). Chemical stabilization of the phycocyanin from cyanobacterium *Spirulina platensis*. *Journal of Biotechnology*.

[100] Telford WG, Moss MW, Morseman JP, Allnutt FCT (2001). Cryptomonad algal phycobiliproteins as fluorochromes for extracellular and intracellular antigen detection by flow cytometry. *Cytometry*.

[101] Sode K, Horikoshi K, Takeyama H, Nakamura N, Matsunaga T (1991). On-line monitoring of marine cyanobacterial cultivation based on phycocyanin fluorescence. *Journal of Biotechnology*.

[102] Izydorczyk K, Tarczynska M, Jurczak T, Mrowczynski J, Zalewski M (2005). Measurement of phycocyanin fluorescence as an online early warning system for cyanobacteria in reservoir intake water. *Environmental Toxicology*.

[103] Simis SGH, Peters SWM, Gons HJ (2005). Remote sensing of the cyanobacterial pigment phycocyanin in turbid inland water. *Limnology and Oceanography*.

[104] Badakere SS, Chablani AT, Bhatia HM, Perjwani DP, Mehta BC (1982). *Recent Trends in Immunology*.

[105] Belay A (2002). The potential application of *Spirulina (Arthrospira)* as a nutritional and therapeutic supplement in health management. *The Journal of the American Nutraceutical Association*.

[106] Liu Y, Xu L, Cheng N, Lin L, Zhang C (2000). Inhibitory effect of phycocyanin from *Spirulina platensis* on the growth of human leukemia K562 cells. *Journal of Applied Phycology*.

[107] Cherng S-C, Cheng S-N, Tarn A, Chou T-C (2007). Anti-inflammatory activity of C-phycocyanin in lipopolysaccharide-stimulated RAW 264.7 macrophages. *Life Sciences*.

[108] Sathyasaikumar KV, Swapna I, Reddy PVB (2007). Co-administration of C-phycocyanin ameliorates thioacetamide-induced hepatic encephalopathy in Wistar rats. *Journal of the Neurological Sciences*.

[109] Dasgupta T, Banerjee S, Yadav PK, Rao AR (2001). Chemomodulation of carcinogen metabolising enzymes, antioxidant profiles and skin and forestomach papillomagenesis by *Spirulina platensis*. *Molecular and Cellular Biochemistry*.

[110] Upasani CD, Balaraman R (2003). Protective effect of *Spirulina* on lead induced deleterious changes in the lipid peroxidation and endogenous antioxidants in rats. *Phytotherapy Research*.

[111] Bermejo P, Piñero E, Villar ÁM (2008). Iron-chelating ability and antioxidant properties of phycocyanin isolated from a protean extract of *Spirulina platensis*. *Food Chemistry*.

[112] Huang Z, Guo BJ, Wong RNS, Jiang Y (2007). Characterization and antioxidant activity of selenium-containing phycocyanin isolated from *Spirulina platensis*. *Food Chemistry*.

[113] McCarty MF (2007). ‘Iatrogenic Gilbert syndrome’—a strategy for reducing vascular and cancer risk by increasing plasma unconjugated bilirubin. *Medical Hypotheses*.

[114] Wang H, Liu Y, Gao X, Carter CL, Liu Z-R (2007). The recombinant *β* subunit of C-phycocyanin inhibits cell proliferation and induces apoptosis. *Cancer Letters*.

[115] Leung P, Lee H, Kung Y, Tsai M, Chou T (2013). Therapeutic effect of C-phycocyanin extracted from blue green algae in a rat model of acute lung injury induced by lipopolysaccharide. *Evidence-Based Complementary and Alternative Medicine*.

